# Enhancing Blockade of *Plasmodium falciparum* Erythrocyte Invasion: Assessing Combinations of Antibodies against PfRH5 and Other Merozoite Antigens

**DOI:** 10.1371/journal.ppat.1002991

**Published:** 2012-11-08

**Authors:** Andrew R. Williams, Alexander D. Douglas, Kazutoyo Miura, Joseph J. Illingworth, Prateek Choudhary, Linda M. Murungi, Julie M. Furze, Ababacar Diouf, Olivo Miotto, Cécile Crosnier, Gavin J. Wright, Dominic P. Kwiatkowski, Rick M. Fairhurst, Carole A. Long, Simon J. Draper

**Affiliations:** 1 The Jenner Institute, University of Oxford, Oxford, United Kingdom; 2 Laboratory of Malaria and Vector Research, NIAID, NIH, Rockville, Maryland, United States of America; 3 KEMRI Centre for Geographic Medicine Research, Kilifi, Kenya,; 4 MRC Centre for Genomics and Global Health, University of Oxford, Oxford, United Kingdom; 5 Mahidol-Oxford Tropical Medicine Research Unit, Mahidol University, Bangkok, Thailand; 6 Wellcome Trust Sanger Institute, Hinxton, Cambridge, United Kingdom; 7 Wellcome Trust Centre for Human Genetics, University of Oxford, Oxford, United Kingdom; London School of Hygiene and Tropical Medicine, United Kingdom

## Abstract

No vaccine has yet proven effective against the blood-stages of *Plasmodium falciparum*, which cause the symptoms and severe manifestations of malaria. We recently found that PfRH5, a *P. falciparum*-specific protein expressed in merozoites, is efficiently targeted by broadly-neutralizing, vaccine-induced antibodies. Here we show that antibodies against PfRH5 efficiently inhibit the *in vitro* growth of short-term-adapted parasite isolates from Cambodia, and that the EC_50_ values of antigen-specific antibodies against PfRH5 are lower than those against PfAMA1. Since antibody responses elicited by multiple antigens are speculated to improve the efficacy of blood-stage vaccines, we conducted detailed assessments of parasite growth inhibition by antibodies against PfRH5 in combination with antibodies against seven other merozoite antigens. We found that antibodies against PfRH5 act synergistically with antibodies against certain other merozoite antigens, most notably with antibodies against other erythrocyte-binding antigens such as PfRH4, to inhibit the growth of a homologous *P. falciparum* clone. A combination of antibodies against PfRH4 and basigin, the erythrocyte receptor for PfRH5, also potently inhibited parasite growth. This methodology provides the first quantitative evidence that polyclonal vaccine-induced antibodies can act synergistically against *P. falciparum* antigens and should help to guide the rational development of future multi-antigen vaccines.

## Author Summary

Malaria is the most devastating parasitic disease of humans, resulting in an estimated 0.6–1 million deaths per year. The symptoms of malaria are caused when merozoites invade and replicate within red blood cells, and therefore a vaccine which induced antibodies that effectively prevent this invasion process would be a major step towards the control of the disease. However, development of such a vaccine has proved extremely challenging. A major roadblock has been the probable need for extremely high levels of antibodies to achieve vaccine efficacy. We have now shown that antibodies against the merozoite protein PfRH5 are able to neutralize the invasion of red blood cells by malaria parasites at concentrations that are significantly lower than for antibodies against PfAMA1 – the previous leading blood-stage malaria vaccine target. This neutralization was observed in both laboratory-adapted parasite lines and in five different parasite isolates from Cambodian patients with malaria. Furthermore, we found that by combining antibodies against PfRH5 with antibodies against certain other merozoite antigens we could achieve synergistic neutralization of parasites, further lowering the amount of antibody needed to be induced by a vaccine. The development of vaccines encoding the PfRH5 antigen in combination with a second target may thus be the best way to achieve the long-sought after goal of an efficacious blood-stage malaria vaccine. Moreover, the methodology described here to assess the ability of antibodies against different targets to synergize should greatly aid the future rational design of improved vaccine candidates.

## Introduction

The pathogenic blood-stages of the *Plasmodium falciparum* life-cycle, whereby merozoites invade and multiply within erythrocytes, cause the symptoms and severe manifestations of malaria – a disease resulting in 600,000 to 1.2 million deaths annually 1,2]. There is thus a pressing need for a highly effective vaccine, but clinical trials of leading blood-stage antigens such as *P. falciparum* apical membrane antigen 1 (PfAMA1) and merozoite surface protein 1 (PfMSP1) have proven disappointing. No Phase IIa/b trial of a blood-stage vaccine has yet reported significant efficacy with regard to a primary endpoint 3]. Efforts have been hampered by antigenic polymorphism rendering many candidate vaccines strain-specific, and the apparent need for very high antibody levels to achieve protection 4–9]. These problems may be overcome by identifying conserved antigens that are more susceptible to vaccine-induced antibodies, and/or the identification of at least two antigens that elicit synergistically-acting antibodies, thus lowering the total level of vaccine-induced antibody required to achieve protection 10].

Multiple ligand-receptor interactions are involved in merozoite invasion of erythrocytes, and it is possible that these interactions may be blocked by vaccine-induced antibodies. In particular, members of the *P. falciparum* reticulocyte-binding homologue (PfRH) and erythrocyte binding antigen (PfEBA) protein families have been proposed as vaccine targets, as these are thought to mediate attachment to and invasion of erythrocytes 11]. However, with the notable exception of PfRH5, genetic deletion of any one of the PfEBA or PfRH proteins is non-lethal in cultured parasite lines 12], suggesting a level of redundancy between these proteins. For example, deletion of the gene encoding PfEBA175 results in up-regulation of the gene encoding PfRH4 in parasite lines that previously did not rely on this ligand 13]. In contrast, repeated attempts to knock out the gene encoding PfRH5 have failed 14,15], and the interaction between PfRH5 and its erythrocyte receptor basigin seems to be essential for erythrocyte invasion 16]. Recently, we found that antibodies induced by viral-vectored vaccines encoding full-length PfRH5 potently inhibit the *in vitro* growth of *P. falciparum* 17]. This inhibition was observed in all laboratory-adapted parasite lines tested, suggesting that PfRH5 is a conserved antigen and potentially effective vaccine target. In contrast, well-studied *P. falciparum* blood-stage antigens such as PfAMA1 and PfMSP1 are highly polymorphic 18–21], and when used as single-allele vaccine antigens elicit allele-specific antibody responses that may be ineffective against diverse, naturally-circulating parasites 9]. Since full-length PfRH5 seems to circumvent the problems associated with antigen polymorphism, and performs no worse than PfAMA1 against vaccine-homologous parasites *in vitro* 17], this antigen is a leading candidate for inclusion in new blood-stage vaccines that aim to induce merozoite-neutralizing antibodies.

Despite the promise shown in pre-clinical studies by vaccines encoding full-length PfRH5 alone 17], it also remains essential to continue optimizing pre-clinical, next-generation vaccine candidates. One strategy is to assess multi-antigen vaccines for their potential to induce antibodies that act synergistically, in order to achieve the highest levels of parasite neutralization for any given level of vaccine-induced antibody. Several studies have investigated the effects of mixing antibodies against multiple merozoite antigens on parasite neutralization. For example, co-immunizing rabbits with PfEBA175, PfRH2 and PfRH4 elicits an antibody repertoire that is more potent in inhibiting parasite growth than antibodies elicited by any single one of these antigens 22]. It has also been reported that antibodies against GPI-anchored micronemal protein (PfGAMA) and PfEBA175 additively inhibit parasite growth, while antibodies against PfGAMA and PfAMA1 did not produce additive effects 23]. Ord *et al*. reported some degree of synergy between antibodies against PfRH5 and PfEBA175, but only at low antibody concentrations 24]. Furthermore, antibodies against *P. falciparum* RH5 interacting protein (PfRipr), which forms a complex with PfRH5, inhibit the growth of multiple *P. falciparum* lines additively when combined with antibodies against PfEBA175, PfRH2 and PfRH4 25]. However, none of these studies quantitatively assessed the interactions between antibody specificities in a manner that distinguishes additive from synergistic effects. Methodology for such assessments is well-advanced in the field of pharmacology, notably in antimicrobial chemotherapy development, but has not yet been applied to combinations of vaccine-induced antibodies against different antigens.

By definition, vaccines inducing antibody combinations that produce synergistic (as opposed to additive) effects need to induce lower levels of antibody to achieve an equivalent level of parasite neutralization. Given the inherent difficulties in including more than one antigen in a vaccine 26], a careful and rational assessment of potential combinations is required. Here we investigated parasite neutralization by purified IgG against PfRH5 in combination with IgG against seven other merozoite antigens. We identified several synergistic antibody interactions, validating a new quantitative methodology for the rational assessment of multi-antigen, blood-stage vaccines and providing fresh insights into immunity to *P. falciparum*. We also show that antibodies against full-length PfRH5 are highly effective in neutralizing short-term-adapted parasite isolates from Cambodian patients with malaria, as well as laboratory-adapted parasite lines 17], demonstrating the critical role of PfRH5 in the growth of parasite isolates from a malaria-endemic region. Furthermore, antibodies against PfRH5 are more potent than those against the leading blood-stage vaccine candidate PfAMA1, as indicated by a lower concentration of antigen-specific IgG required to give 50% growth inhibitory activity (GIA EC_50_). Vaccine-induced *in vitro* GIA has been associated with protection in multiple non-human primate challenge studies 4,27,28], but this may represent a form of non-natural malaria immunity and in the absence of a protective vaccine has yet to be confirmed as a protective mechanism in humans 29,30]. Nevertheless, our data provide additional evidence that PfRH5 is presently the most promising candidate antigen for inclusion in a GIA-inducing blood-stage vaccine against *P. falciparum* malaria.

## Results

### Quantification of EC_50_ of antigen-specific IgG in GIA assays

GIA is routinely assessed with total IgG purified from immunized animals, only a small fraction of which is specific for the immunogen. A novel method for the measurement of antigen-specific polyclonal antibody concentrations has recently been described 31]. This method, termed calibration-free concentration analysis (CFCA), depends on measurement of antibody binding rates by surface plasmon resonance (SPR), under conditions in which antibody concentration is the rate-limiting factor. Using CFCA, we quantified the antigen-specific antibody EC_50_ values of IgG purified from the sera of rabbits immunized with viral vectors encoding both PfRH5 and PfAMA1. We have established that results obtained with this method correlate closely with measurements of antibody concentrations obtained using a standardized ELISA 32], and with spectrometer-determined concentrations of a range of anti-PfRH5 mouse mAbs (ADD *et al.*, manuscript in preparation).

**Figure 1:**
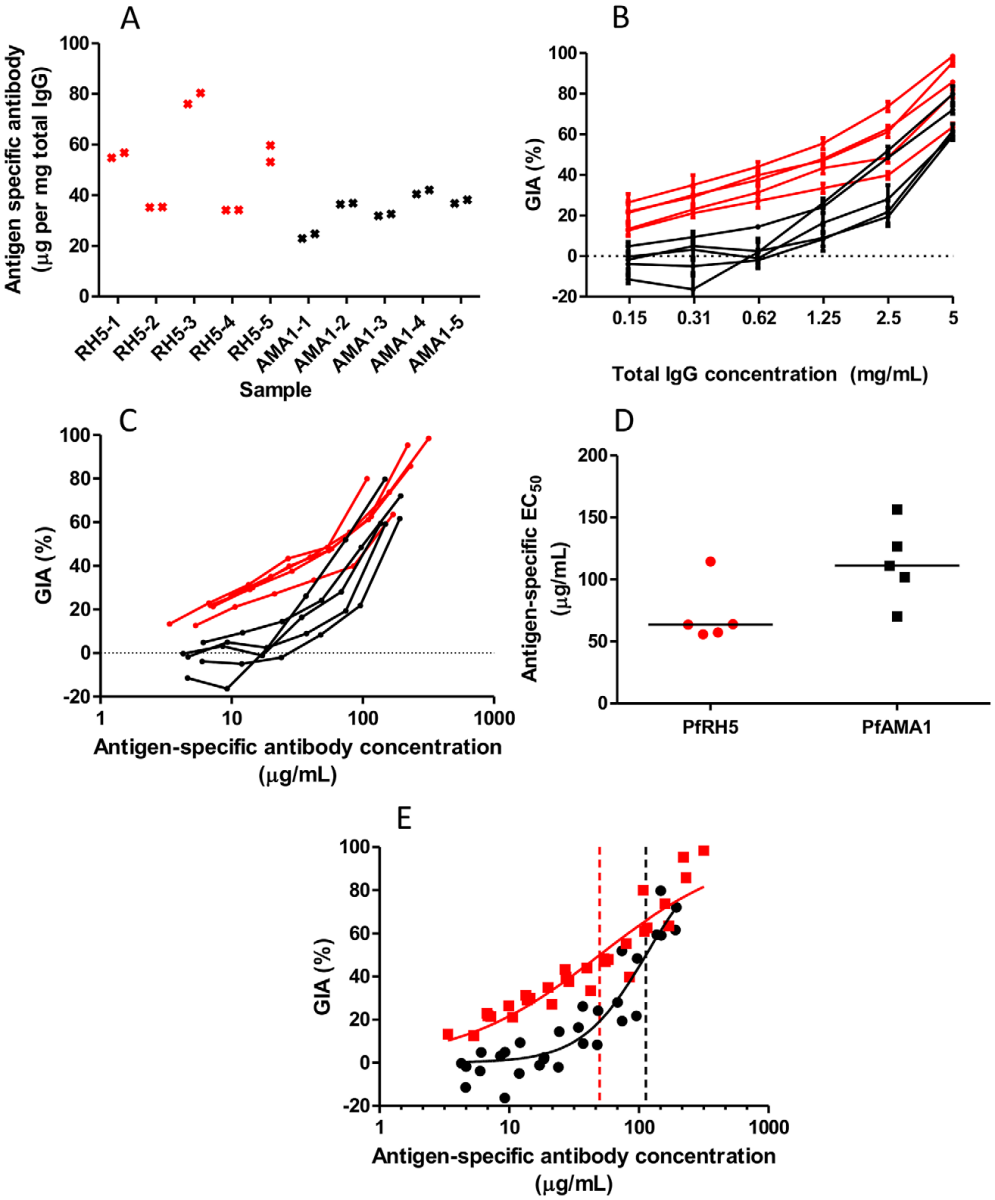
Measurement of GIA EC_50_ of antigen-specific anti-PfRH5 and anti-PfAMA1 polyclonal rabbit antibodies. **Panel A** - CFCA-measured antigen-specific antibody as a proportion of total IgG (measured by spectrometry) for each of ten rabbits. Individual points indicate mean of three measurements. **Panel B** - GIA vs. total IgG concentration, with lines connecting data for each of five PfRH5-vaccinated rabbits (red) and five PfAMA1-vaccinated rabbits (black). Each point is the mean of three replicate wells in two independent experiments, i.e. *n* = 6. Error bars indicate SEM. **Panel C** - GIA (from the experiments depicted in panel **B**) vs. antigen-specific antibody concentration (calculated for each sample using the data in panel **A**), for each of five PfRH5-vaccinated rabbits (red) and five PfAMA1-vaccinated rabbits (black). Each point is the mean of triplicate wells in two independent experiments. **Panel D** - antigen-specific antibody EC_50_ values for PfRH5 and PfAMA1, calculated by interpolation from the data in panel **C**. Individual data-points and the median are shown. **Panel E** - Dose-response curve fitted to all GIA vs. antigen-specific antibody concentration data for the 3D7 parasite clone (multiple IgG dilutions for each of five rabbits for PfRH5 and PfAMA1). Dashed vertical lines indicate the fitted EC_50_ value for anti-PfRH5 (red) or anti-PfAMA1 (black) IgG. Each GIA value is the mean of triplicate wells in each of two experiments (*n* = 6). Red indicates anti-PfRH5 samples; black indicates anti-PfAMA1 samples.

Antigen-specific antibody concentrations were measured by CFCA in total IgG samples purified from each of ten rabbits, five vaccinated with PfRH5 and five with PfAMA1 (Figure 1A). We then independently determined the EC_50_ of antigen-specific antibody from each rabbit in GIA assays conducted with total IgG against the 3D7 *P. falciparum* clone (Figure 1B–D). We found the median antigen-specific EC_50_ to be 111 µg/mL for PfAMA1 (range 70–156 µg/mL, 95% CI for mean 74–152 µg/mL). This GIA EC_50_ value is close to that previously reported for 3D7 PfAMA1-specific rabbit antibody (70 µg/mL [95% CI for mean 50–100 µg/mL], obtained using affinity-purified anti-PfAMA1 antibody 6]), further supporting the validity of the CFCA method. We observed a median antigen-specific antibody EC_50_ of 64 µg/mL for PfRH5 (range 55–114 µg/mL, 95% CI for mean 46–101 µg/mL), around 40% lower than that observed for PfAMA1, although this did not reach statistical significance (Figure 1D, *P* = 0.055, Mann-Whitney U test).

We obtained a second estimate of the antigen-specific antibody EC_50_ for each antigen against the 3D7 parasite clone by pooling data from all five rabbits and fitting a dose-response curve by non-linear least squares regression (Figure 1E). As expected, these results were similar to those obtained from the individual samples' median EC_50_ values: 113 µg/mL (95% CI 97–132 µg/mL) for PfAMA1 and 50 µg/mL (95% CI 41–60 µg/mL) for PfRH5. This analysis shows significant differences in both the EC_50_ (lower with anti-PfRH5 IgG) and the slope of the curves (steeper with anti-PfAMA1 IgG) (*P*<0.0001 by extra sum-of-squares F-test).

It thus appears that the antigen-specific rabbit antibody EC_50_ for anti-PfRH5 IgG is approximately half of that observed for anti-PfAMA1 IgG against the vaccine-homologous 3D7 parasite clone.

### Antibodies against PfRH5 are highly effective against *P. falciparum* isolates obtained from Cambodian patients with malaria

**Figure 2:**
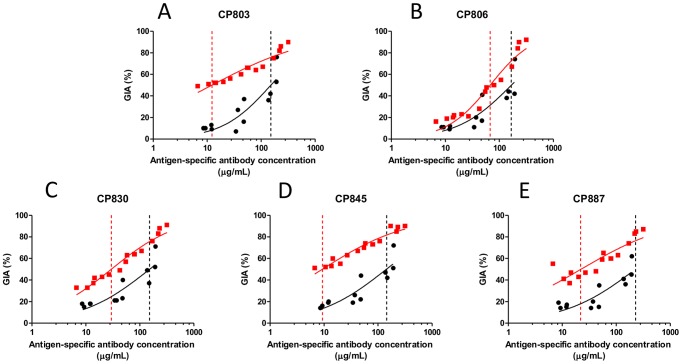
Antigen-specific EC_50_ estimation for anti-PfRH5 and anti-PfAMA1 IgG against short-term-adapted Cambodian parasite isolates. Dose-response curves were fitted to all GIA versus antigen-specific antibody concentration data for **A**) CP803, **B**) CP806, **C**) CP830, **D**) CP845 and **E**) CP887 (multiple IgG dilutions for each of five rabbits for PfRH5 and each of four rabbits for PfAMA1). Dashed vertical lines indicate the fitted EC_50_ value for anti-PfRH5 IgG (red) or anti-PfAMA1 (black) IgG for that isolate. Each value is the mean of three wells in a single experiment. Red indicates anti-PfRH5 samples; black indicates anti-PfAMA1 samples.

We previously established the potency of anti-PfRH5 antibodies against laboratory-adapted *P. falciparum* lines 17]. In order for PfRH5 to be a viable vaccine candidate antigen, anti-PfRH5 antibodies must be effective against naturally-circulating parasite isolates that cause malaria. To investigate this possibility, we performed GIA assays using five *P. falciparum* isolates that were obtained directly from Cambodian patients with malaria. 18-microsatellite analysis of these parasite isolates indicated that CP806, CP830, CP845 and CP887 were clonal (or clone-predominant) and that CP803 was multi-clonal (RMF *et al*., unpublished). IgG from rabbits immunized with PfRH5 was highly effective against all five parasite isolates (Figure 2 and Figure S1A–E). Antigen-specific IgG EC_50_ values were estimated for anti-PfRH5 and anti-PfAMA1 IgG samples against each parasite isolate, as performed previously for the 3D7 parasite clone. For each parasite isolate, these values were significantly lower for anti-PfRH5 than for anti-PfAMA1 IgG samples (Figure 2, Figure S1F and Table 1; *P*<0.05 by Mann-Whitney U test).

**Table 1:** GIA EC_50_ values of anti-PfRH5 and anti-PfAMA1 IgG against various Cambodian parasite isolates and the 3D7 parasite clone.

Parasite	EC_50_ (mg/mL total IgG, anti-PfAMA1 IgG)	EC_50_ (mg/mL total IgG, anti-PfRH5 IgG)	EC_50_ (µg/mL PfAMA1-specific IgG)	EC_50_ (µg/mL PfRH5-specific IgG)
**CP803**	*6.2*	*0.3*	151	12
**CP806**	*7.8*	1.5	167	68
**CP830**	*5.3*	*0.8*	152	30
**CP845**	*5.5*	*0.2*	143	9
**CP887**	*7.9*	*0.7*	*227*	22
**3D7**	3.1 (2.27–3.81)	1.5 (0.95–2.01)	113	50

Total IgG EC_50_ value for Cambodian parasite isolates is the median of observed values for five individual rabbits; values in italics are extrapolated from observed values. Antigen-specific antibody EC_50_ values are derived from fitting of a single curve to all available GIA data points for each antigen and each parasite (as depicted in Figure 1E and Figure 2). For comparison, estimated EC_50_ values of the total and antigen-specific IgG against the 3D7 parasite clone are also shown. 3D7 antigen-specific IgG values are as calculated in Figure 1E, whereas total IgG values are a mean, with 95% CI in parentheses, from eight independent experiments that have estimated the total IgG EC_50_ of anti-PfRH5 and anti-PfAMA1, with a total of five immunized rabbits for each antigen (Figure 1B, Figure 4A–B, Reference 17]).

Sequencing of the PfRH5 gene in 18 laboratory-adapted parasite lines has previously shown a total of ten non-synonymous mutations 15]. Polyclonal IgG against PfRH5 (3D7 clone) remained effective against parasite lines differing from 3D7 at each of these loci 17]. Whole-genome sequencing of the Cambodian parasite isolates revealed non-reference alleles at two non-synonymous SNPs in the PfRH5 gene that were not identified in the sequencing of laboratory-adapted parasite lines (Table 2). In a global genomic epidemiology study 33] comprising 227 isolates from South East Asia, Africa and Papua New Guinea, these variants were found to be relatively common in South East Asia (allele frequencies of 0.28 and 0.30). However, no significant association was observed between the presence of these novel mutant alleles and the EC_50_ of anti-PfRH5 IgG against any of the Cambodian parasite isolates. Moreover, there were no other PfRH5 SNPs with an allele frequency greater than 0.05 in any of the aforementioned geographical regions (Table 2); since PfRH5 had sufficient read coverage in most isolates, it is unlikely that any important SNP was overlooked due to poor genotyping (OM and DPK, unpublished). Hence, we expect novel SNP polymorphisms that are not currently catalogued to be at very low frequency in the three geographical areas sampled by the study. The functional importance of PfRH5 therefore appears to be conserved across both laboratory-adapted parasite lines and parasite isolates from multiple malaria-endemic regions. Crucially, these data suggest that a vaccine against PfRH5 may be able to induce antibodies that neutralize diverse, naturally-circulating *P. falciparum* parasites.

**Table 2:** PfRH5 genotypes in parasites isolated from Cambodia.

Polymorphism	Field isolates tested in GIA in current study					Non-3D7 allele frequency			
	CP803	CP806	CP830	CP845	CP887	SEA	AFR	PNG	Lab
E48K	E	E	E	E	E	0	0	0	0.09
E69E (S)									
N88D	N	N	N	N	N	0	0.01	0	0
*Y147H*	*H*	*H*	*Y*	*Y*	*H*	*0.28*	*0.09*	*0.05*	*0*
*H148D*	*D*	*D*	*H*	*H*	*D*	*0.3*	*0.1*	*0.05*	*0*
**S197Y**	**S&Y**	**?**	**S**	**S&Y**	**Y**	**0.54**	**0**	**0.38**	**0.23**
**C203Y**	**Y**	**Y**	**C**	**C&Y**	**Y**	**0.62**	**0.79**	**0.9**	**0.77**
I204K,R	I	I	I	I	I	0	0	0	0.09
A233E	A	A	A	A	A	0	0	0.05	0
N347Y,D	N	N	N	N	N	0	0	0	0.09
Y358F	Y	Y	Y	Y	Y	0	0	0	0.09
E362D	E	E	E	E	E	0	0.01	0	0.05
I364I (S)									
H365N	H	H	H	H	H	0	0.01	0	0
V371I	V	V	V	V	V	0	0.05	0	0
I407V	I	I	I	I	I	0	0.03	0	0.05
**I410M**	**I**	**I**	**M**	**I**	**I**	**0.35**	**0**	**0.1**	**0.09**
K429N	K	K	K	K	K	0	0	0	0.14
Q477H	Q	Q	Q	Q	Q	0	0.01	0	0
I493V	I	I	I	I	I	0	0	0	0

Amino acids are represented by single letter codes. ‘Polymorphism’ denotes the reference (3D7) allele, amino acid number and non-reference allele. The presence of two amino acids at a single locus indicates a mixed genotype. ‘?’ indicates unknown. ‘Non-3D7 allele frequency’ denotes the proportion of sequenced loci with a non-3D7 allele at that locus: Lab = 22 laboratory lines previously sequenced 14–16]. For comparison, we show allele frequencies estimated by genome sequencing for three major endemic regions 33], using the online database provided at http://www.malariagen.net/data. SEA = South East Asia (81 samples from Thailand and Cambodia), AFR = Africa (125 samples from Kenya, Mali and Burkina Faso) and PNG = Papua New Guinea (21 samples).

(S) indicates synonymous SNPs; italic text indicates SNPs not previously identified in laboratory-adapted parasite lines and with non-reference allele frequency >5% in at least one population of parasite isolates; bold text indicates SNPs with non-reference allele frequency >5% of laboratory-adapted parasite lines and parasite isolates; for all other SNPs, the non-reference allele frequency is ≤5% in all sequenced populations of parasite isolates.

### Antibodies against PfRH5 can act synergistically with antibodies against other merozoite antigens

The concentration of vaccine-induced antibody required to neutralize parasites will be lower if antibodies against multiple antigens can act synergistically. We next systematically combined purified anti-PfRH5 rabbit IgG in GIA assays with purified rabbit IgG against seven other merozoite antigens – PfAMA1, PfMSP1 (a construct containing the conserved blocks of sequence 1, 3, 5 and 12 followed by both of the dimorphic forms of the 42 kDa C-terminus, MSP1_42_), PfEBA175, PfRH2, PfRH4, Pf38 and Pf Rhoptry Associated Protein 3 (PfRAP3). To detect synergy, we measured the GIA effect of a fixed concentration of anti-PfRH5 IgG with or without the addition of a range of concentrations of IgG against the other merozoite antigens. For each combination of antibodies, we calculated the predicted GIA that would be achieved by the two components having an independent, additive effect by using the definition of Bliss additivity 34]. We then compared the observed and predicted GIA for each combination, with statistically significant deviations indicating non-independence, either synergy (greater GIA effect than the predicted value) or sub-additivity (weaker effect than the predicted value).

**Figure 3:**
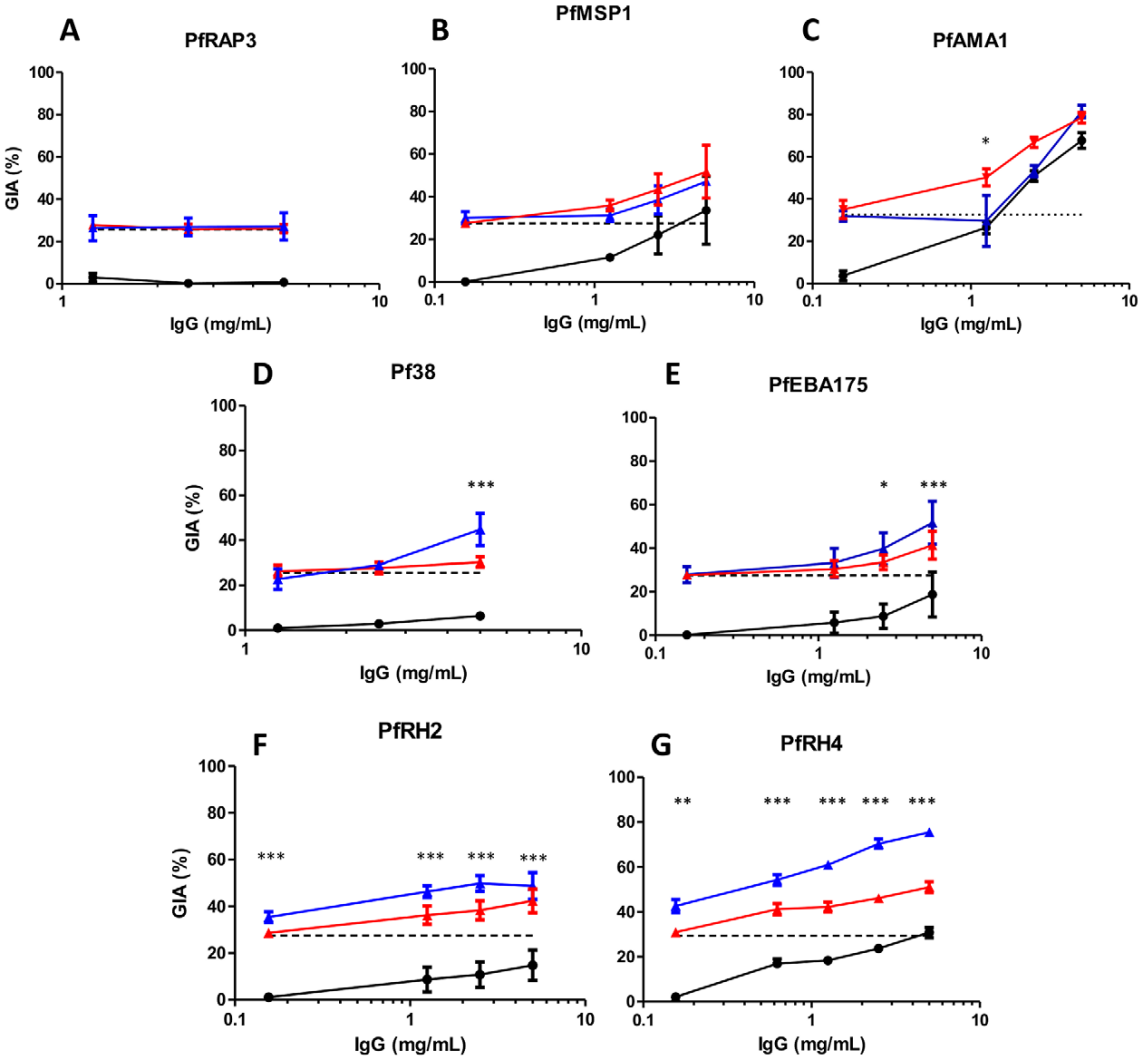
GIA effects of anti-PfRH5 IgG in combination with polyclonal antibody specific for other merozoite antigens. Percentage GIA against the 3D7 parasite clone over increasing concentrations of total purified IgG from rabbits immunized with **A**) PfRAP3, **B**) PfMSP1, **C**) PfAMA1, **D**) Pf38, **E**) PfEBA175, **F**) PfRH2 and **G**) PfRH4, with (blue line) or without (solid black line) the addition of a fixed low concentration of PfRH5-immunized rabbit IgG (0.156 mg/mL) which, when used alone, gives approximately 25% GIA (dashed black line). Predicted additive effects were calculated according to Bliss independence (see Materials and Methods) and illustrated as the red line on each graph. Data points represent the mean of triplicates from two independent experiments. Bars indicate SEM for all six replicates over two experiments. Asterisks indicate that the predicted and observed values differed significantly (*P<0.05; **P<0.01; ***P<0.001, 2-way ANOVA with Bonferroni post-hoc testing).

There was no evidence of a synergistic effect against the 3D7 parasite clone when IgG against PfRH5 was combined with IgG against PfRAP3, PfMSP1 or PfAMA1 (Figure 3A–C). No GIA was observed at any tested concentration of anti-PfRAP3 IgG, and no differences were observed between the level of GIA achieved by anti-PfRH5 IgG alone or in combination with anti-PfRAP3 IgG (Figure 3A). Low levels of GIA were achieved by anti-PfMSP1 IgG, but the addition of anti-PfRH5 IgG produced values similar to those predicted by Bliss independence, indicating an additive, independent effect of these antibodies (Figure 3B). Combining anti-PfRH5 and high levels of anti-PfAMA1 IgG (5 mg/mL) also resulted in additive GIA similar to that predicted by Bliss independence. Lower concentrations of anti-PfAMA1 (≤2.5 mg/mL) resulted in less GIA than the predicted additive, reaching statistical significance at 1.25 mg/mL (*P*<0.05; Figure 3C). While this could be described as an antagonistic interaction, GIA was never lower than that achieved by the more potent individual component; we thus describe this interaction as sub-additive rather than antagonistic.

In contrast, combining anti-PfRH5 IgG with anti-Pf38 or anti-PfEBA175 IgG produced synergistic effects at high concentrations of the latter antibodies (Figure 3D,E). The observed GIA was significantly higher than the predicted values at 5 mg/mL of anti-Pf38 IgG (*P*<0.001, Figure 3D), and at both 2.5 mg/mL (*P*<0.05) and 5 mg/mL of anti-PfEBA175 IgG (*P*<0.001, Figure 3E). Lower concentrations of anti-Pf38 and anti-PfEBA175 IgG neither achieved detectable GIA when used alone, nor resulted in a significant change in GIA from that achieved by 0.156 mg/mL of anti-PfRH5 IgG when used in a mixture.

Strikingly, when anti-PfRH5 IgG was combined with IgG against PfRH2 and PfRH4, a clear synergistic effect was observed. Here, every concentration of anti-PfRH2 or anti-PfRH4 IgG tested (ranging from 0.156–5 mg/mL) produced a statistically significant synergistic effect when combined with anti-PfRH5 IgG (*P*<0.01; Figure 3F,G). These data indicate that the greatest synergistic activity may be achieved when combining anti-PfRH5 IgG with antibodies against other PfRH family members.

### Quantitative assessment of synergy

**Figure 4:**
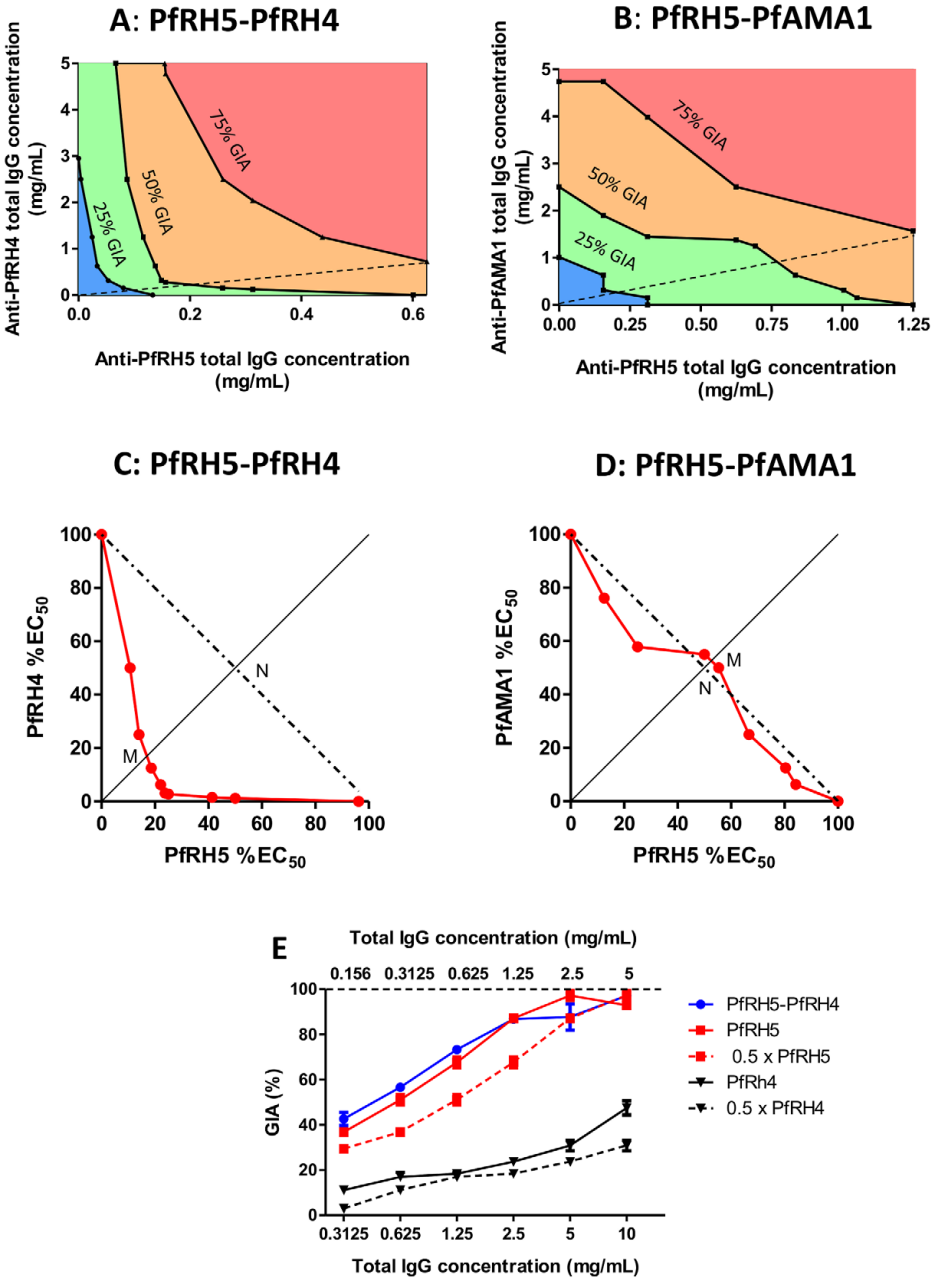
Contour plots and isobolograms of GIA achieved by anti-PfRH5 IgG in combination with either anti-PfRH4 or anti-PfAMA1 IgG. **Panel A,B**–Contour plots of GIA versus concentration of total IgG combined from rabbits immunized with either PfRH5 or PfRH4 (A), or with either PfRH5 or PfAMA1 (**B**). Each experiment was conducted independently. Black lines are contours linking anti-PfRH5 and anti-PfRH4 IgG combinations inducing 25%, 50% and 75% GIA (as labelled), obtained by interpolation between observed GIA values. Shaded area indicates 0–25% GIA (blue), 25–50% GIA (green), 50–75% GIA (orange), and 75–100% GIA (pink). Thin diagonal dashed line from origin indicates line of equal concentration of IgG from each component. **Panel C,D** – 50% GIA isobologram for anti-PfRH5 and anti-PfRH4 IgG (**C**) and anti-PfRH5 and anti-PfAMA1 IgG (**D**) combinations. Red line links the observed combination of anti-PfRH5 IgG and either anti-PfRH4 or anti-PfAMA1 IgG that induced 50% GIA, plotted on axes of anti-PfRH5 and anti-PfRH4/anti-PfAMA1 IgG concentration expressed as percentage of the EC_50_. Dashed line illustrates 50% contour predicted if anti-PfRH5 IgG and the other antibody are Loewe additive. Diagonal x = y line from origin links points at which anti-PfRH5 and anti-PfRH4/PfAMA1 IgG concentrations (as proportion of EC_50_) are equal; the letters M and N indicate the line intersections used to calculate Hewlett's synergy index. **Panel E** – GIA attained by mixing equal concentrations of anti-PfRH5 with anti-PfRH4 IgG (blue line), plotted against the total IgG concentration in the well shown on the lower x-axis (i.e. twice the concentration of each individual component). The solid red line indicates the GIA effect when anti-PfRH5 IgG is used alone at the concentrations on the lower x-axis (i.e. twice the concentration of anti-PfRH5 IgG in the antibody mixture), and the dashed red line indicates the GIA effect of anti-PfRH5 IgG alone at the concentration shown on the upper dashed x-axis (i.e. the concentration of anti-PfRH5 IgG present in the antibody mixture). The solid and dashed black lines indicate the same relationship for anti-PfRH4 IgG.

3D-surface/contour plots and isobolograms provide quantitative measurements of synergy by comparing the quantity of agents required to achieve a certain effect when mixed, as compared to when used alone 35]. This approach, which makes use of a more robust definition of additivity (termed Loewe additivity 35]), is routinely used to quantify synergy between drugs, and we have applied it here to antibodies specific for merozoite antigens. We performed GIA assays with all combinations of six concentrations (ranging from 0 to 5 mg/mL total IgG) of anti-PfRH5 IgG together with either anti-PfRH4 or anti-PfAMA1 IgG. The constructed plots clearly revealed a concave-rightwards pattern in both the contour plots and isobolograms for the combination of anti-PfRH5 and anti-PfRH4 IgG (Figure 4A,C), in contrast to the parallel contour pattern for anti-PfRH5 and anti-PfAMA1 IgG (Figure 4B,D). For the anti-PfRH5/anti-PfRH4 IgG combination, the 50% isobologram indicates that the concentration of antibody needed to neutralize 50% of parasites is lower than would be predicted if the antibodies were acting independently and thereby producing a Loewe additive effect (Figure 4C). Hewlett's S (a quantitative index of the extent of synergy) had a value of 2.9 (>1 implies synergy). Under the definition of Loewe additivity, half the EC_50_ concentration of anti-PfRH5 IgG combined with half the EC_50_ concentration of anti-PfRH4 IgG would achieve 50% GIA; in this case Hewlett's S value would be 1. The observed Hewlett's S value of 2.9 indicates that the required concentration of each antibody to achieve 50% GIA is approximately 3-fold less than predicted under the assumption of additivity. In contrast, the isobologram for the anti-PfRH5/anti-PfAMA1 IgG combination was close to the line of Loewe additivity, with a value of 0.96 for Hewlett's S (Figure 4D). Thus, despite the appearance of Bliss sub-additivity in our earlier experiment (Figure 3C), anti-PfRH5 and anti-PfAMA1 IgG appear to act virtually additively in combination when assessed using the Loewe definition which accounts for the self-cooperativity of the mixed antibodies. In this context, the Bliss definition of additivity may thus be excessively stringent; nonetheless, the combination of anti-PfRH5 and anti-PfRH4 IgG was clearly synergistic regardless of which method was used to define synergy.

Although the isobologram approach provides clear analysis of synergy, it is perhaps more relevant to the *in vivo* situation to consider the effects of mixing equal concentrations of IgG from animals immunized with different antigens. We examined this for the combination of anti-PfRH5 and anti-PfRH4 IgG which was clearly defined as synergistic in the preceding experiments. This information can be extracted from the data used to construct contour plots (represented by the dashed line in Figure 4A), and are shown in Figure 4E. The effect of combining an equal amount of anti-PfRH5 with anti-PfRH4 IgG was similar to doubling the concentration of anti-PfRH5 IgG, i.e. the 50∶50 combination of IgG was equivalent to the same total amount of IgG against PfRH5 alone. However, the GIA of the combination was clearly superior to that achieved by doubling the concentration of anti-PfRH4 IgG, emphasizing the large differences in potency of the two antibody specificities. Therefore, for very potent antibodies such as anti-PfRH5 IgG, equivalent increases in GIA may be achieved by either synergistic combination with another component or by relatively modest changes in the concentration of the most potent antibody alone. This clearly has important implications for decision-making regarding potential multi-antigen antibody-inducing vaccines.

### Synergy against vaccine-heterologous parasite lines

**Figure 5:**
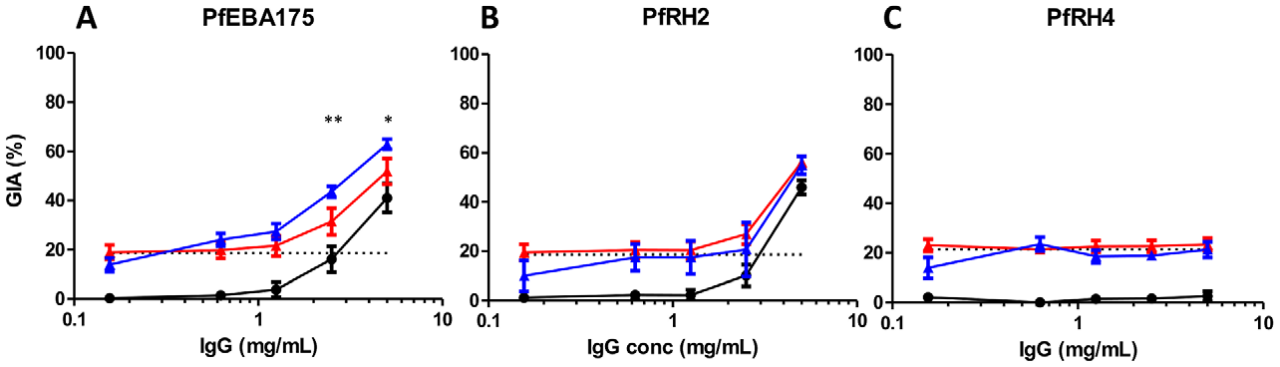
GIA synergies against the vaccine-heterologous FVO parasite clone. Percentage GIA against the FVO parasite clone over increasing gradients of concentrations of IgG from rabbits immunized with **A**) PfEBA175, **B**) PfRH2 or **C**) PfRH4 with (blue line) or without (solid black line) the addition of a fixed low concentration of PfRH5-immunized rabbit IgG (0.156 mg/mL) which, when used alone, gives approximately 20% GIA (dashed black line). Predicted additive effects were calculated according to Bliss independence (see Materials and Methods) and illustrated as the red line on each graph. Data points represent the mean of triplicates from two independent experiments. Bars indicate SEM for all six replicates over two experiments. Asterisks indicate that the predicted and observed values differed significantly (*P<0.05; ** *P*<0.01; 2-way ANOVA with Bonferroni post-hoc testing).

To determine if the additive and synergistic effects we observed against the 3D7 parasite clone are strain-transcending, we used mixtures of anti-PfRH5 IgG with anti-PfEBA175, anti-PfRH2 or anti-PfRH4 IgG in GIA assays using the FVO parasite clone. Of all the laboratory-adapted parasite lines for which the PfRH5 gene sequence has been reported, FVO differs from 3D7 in four amino acids, the most between any pair of parasite lines 15]. We found that when anti-PfRH5 and anti-PfEBA175 IgG were combined, the modest synergistic effect observed against the 3D7 parasite clone was still apparent (Figure 5A). However, when anti-PfRH5 and anti-PfRH2 IgG were combined, only an additive effect was observed (Figure 5B). Moreover, the promising synergy achieved by the combination of anti-PfRH5 and anti-PfRH4 IgG was completely absent, consistent with FVO being reliant on sialic-acid (SA) dependent invasion routes 36] (Figure 5C). Clearly, the rational design of multi-component vaccines that achieve synergistic antibody effects will require assessment of the presence, or absence, of such synergies in the neutralization of multiple parasite lines/isolates.

### Antibodies against the PfRH5 receptor, basigin, also synergize with antibodies against PfRH4 and PfEBA175

We also performed GIA assays with a combination of polyclonal anti-PfRH4 IgG and a mAb against basigin, the PfRH5 erythrocyte receptor, to explore whether they exerted the same synergistic effect against the 3D7 parasite clone as does the combination of anti-PfRH5 and anti-PfRH4 IgG. The anti-basigin mAb TRA-1-85 (1 µg/mL) inhibited the growth of the 3D7 parasite clone by approximately 20%. Strikingly, the addition of 5 mg/mL anti-PfRH4 IgG resulted in almost 100% GIA in a clear synergistic effect. More than 70% GIA was observed when 0.156 mg/mL of anti-PfRH4 IgG (a concentration with virtually no detectable effect when given alone) was mixed with 1 µg/mL TRA-1-85. This synergistic effect was clearly greater than that observed when polyclonal anti-PfRH5 and anti-PfRH4 IgG were combined (Figure 3G).

**Figure 6:**
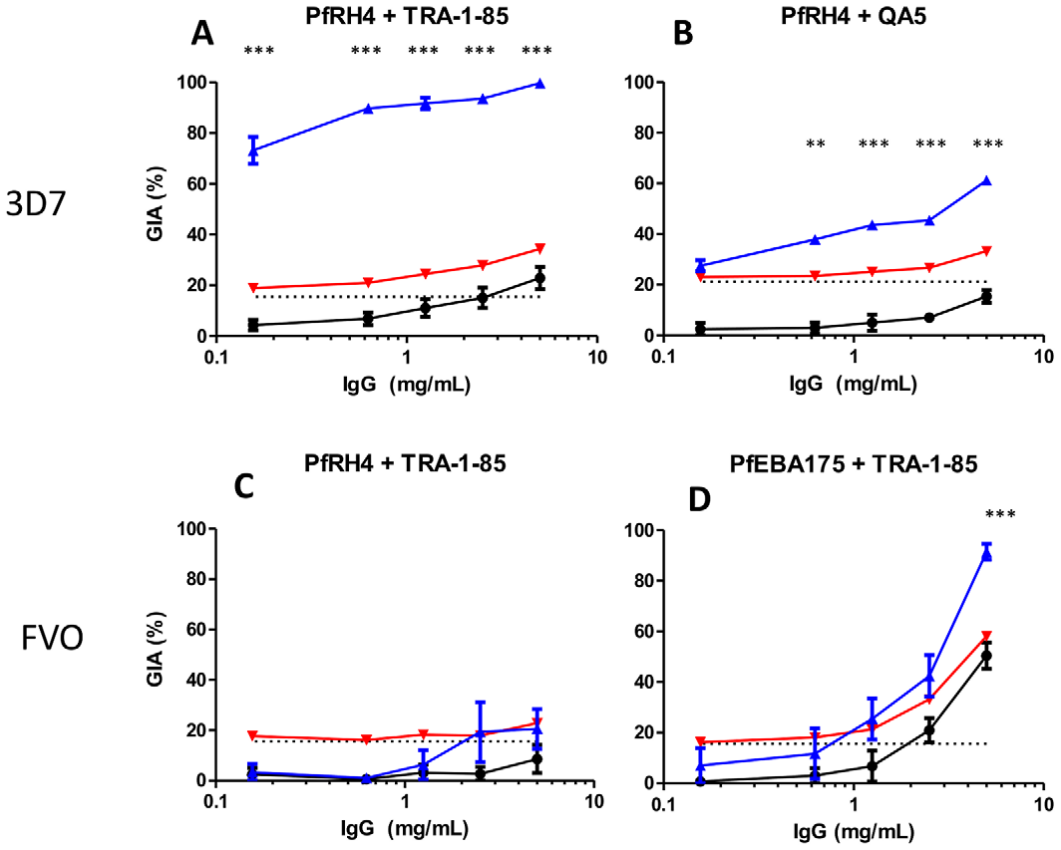
GIA synergies with mixtures of rabbit antisera and mouse monoclonal antibodies. Percentage GIA against 3D7 and FVO parasites using increasing gradients of concentrations of IgG from rabbits immunized with PfRH4 (**Panels A–C**) or PfEBA175 (**Panel D**) with (blue line) or without (solid black line) the addition of a fixed low concentration of either an anti-basigin mAb (TRA-1-85, 1 µg/mL) or anti-PfRH5 mAb (QA5, 10 µg/mL) which, when used alone, give approximately 15–25% GIA (dashed black line). Predicted additive effects were calculated according to Bliss independence (see Materials and Methods) and illustrated as the red line on each graph. Results are mean of two independent experiments for the anti-PfRH4/TRA-1-85 mixture with the 3D7 parasite clone, and one experiment for the other combinations. All measurements were done in triplicate. Bars indicate SEM for all replicates. Asterisks indicate the predicted and observed values differed significantly (** *P*<0.01; ***P<0.001; 2-way ANOVA with Bonferroni post-hoc testing).

To assess whether this strong synergistic effect was parasite-specific, we repeated the experiment with the FVO parasite clone which invades erythrocytes in a SA-dependent manner 36]. In agreement with previous data for the combination of anti-PfRH5 and anti-PfRH4 IgG (Figure 5C), the combination of anti-PfRH4 IgG and TRA-1-85 had no effect above the 15% baseline inhibition given by TRA-1-85 alone at 1 µg/mL, confirming that the synergy of blocking these two pathways is parasite-specific (Figure 6C). We then tested a mixture of TRA-1-85 and anti-PfEBA175 IgG against the FVO parasite clone, which is known to utilize this ligand for invasion 36]. Here, there was clear synergistic inhibition at high concentrations of anti-PfEBA175 IgG, with close to 90% inhibition observed at 5 mg/mL (Figure 6D) – a level of GIA not reached with a combination of polyclonal anti-PfRH5 and anti-PfEBA175 IgG (Figure 5A). The increased GIA seen with anti-basigin antibodies, rather than antibodies against the ligand PfRH5 itself, might be due to the attainment of equilibrium binding of anti-basigin antibodies to the erythrocyte during the assay incubation period, accentuating the effect of the anti-merozoite antibodies which must bind their target when the parasite is briefly exposed to the medium. To test this hypothesis, we combined anti-PfRH4 IgG with the anti-PfRH5 mAb QA5 which blocks the interaction with basigin (ADD *et al.*, manuscript in preparation). Growth inhibition by QA5 is thus likely to be mechanistically similar to that achieved by TRA-1-85. Against the 3D7 parasite clone, this combination (Figure 6B) produced a synergistic effect very similar to the effect seen with polyclonal anti-PfRH5 IgG (Figure 3G), but less dramatic than that with the anti-basigin/anti-PfRH4 IgG combination (Figure 6A), suggesting that the increased synergy observed in the latter combination is likely due to the equilibrium binding of TRA-1-85 to the erythrocyte prior to merozoite invasion.

## Discussion

Development of blood-stage subunit vaccines against *P. falciparum* malaria has proven extremely challenging. The discovery of full-length PfRH5 as a conserved and antibody-susceptible antigen has renewed hope that this challenge may be surmountable. Here and elsewhere 17], we show that multiple laboratory-adapted parasite lines and naturally-circulating parasite isolates are susceptible to anti-PfRH5 IgG, which neutralizes parasites at concentrations that are comparable to or, in many cases, lower than anti-PfAMA1 IgG. Furthermore, it has frequently been suggested that multi-antigen blood-stage vaccines may induce antibodies that act synergistically. Some published data have hinted that such effects may indeed be achievable 22,24]. We have extended upon this previous work, by identifying synergy between antibody combinations using a quantitative methodology that has not, to our knowledge, been previously applied to vaccine development.

The extent of known polymorphism in the PfRH5 gene in laboratory-adapted parasite lines is limited (10 of 526 amino acids) 15]. We have now shown that naturally-circulating *P. falciparum* isolates from Cambodia, as well as laboratory-adapted parasite lines 17], are highly susceptible to anti-PfRH5 antibody. Sequencing of the PfRH5 gene in these Cambodian parasite isolates revealed only two non-synonymous SNPs which have not been previously identified in laboratory-adapted parasite lines, and no PfRH5 SNPs with a minor allele frequency exceeding 5% were identified in sequence data from 227 parasite isolates from diverse geographical areas. Taken together, these data indicate that the parasite isolates used in our assays represent the diversity found in three important malaria endemic regions. Moreover, in addition to the 3D7 parasite clone upon which our PfRH5 immunogen is based, we have now assessed GIA of anti-PfRH5 IgG against five laboratory-adapted parasite lines and five short-term-adapted parasite isolates. We have not observed any apparent relationship between PfRH5 genotype and the EC_50_ of anti-PfRH5 IgG. It seems likely that the extent of polymorphism between 3D7 and other parasite lines (a maximum of four pairwise single amino-acid differences) is insufficient to have a substantial impact upon the binding of polyclonal antibody. The need to interact with basigin 16] and other binding partners, such as PfRipr 25], could impose functional constraints on the possible extent of mutation in PfRH5. Therefore, it may prove challenging for *P. falciparum* to evolve resistance to an efficacious PfRH5 vaccine by means of antigenic drift.

Whilst significantly lower EC_50_ values for anti-PfRH5 IgG than for anti-PfAMA1 IgG were observed in the Cambodian parasite isolates (up to a 17-fold difference), the difference in EC_50_ against the vaccine-homologous 3D7 parasite clone was relatively modest (approximately 2-fold). Given the extremely high titers of anti-PfAMA1 antibodies required to achieve protective effects in non-human primate models of malaria 4], it seems probable that the design of vaccines with lower *in vitro* EC_50_ values will enhance the likelihood of achieving *in vivo* efficacy. Therefore, there may be distinct advantages in identifying an additional antigen that produces synergistic effects when used in combination with anti-PfRH5 IgG.

We observed synergy between antibodies against PfRH invasion ligands, and to a lesser extent, between anti-PfRH5 IgG and antibody to PfEBA175 (F2 region), another well-characterized invasion ligand. The other antibody combinations we tested (notably mixtures of anti-PfRH5 with either anti-PfAMA1 or anti-PfMSP1 IgG) achieved levels of GIA close to those predicted by Bliss additivity (or in the case of PfAMA1 IgG, Loewe additivity). This is consistent with previous work showing a lack of synergy when antibodies against PfAMA1 and the 19 kDa C-terminus of PfMSP1 (PfMSP1_19_) are combined 37,38]. Interestingly, we did not identify antibody combinations resulting in truly antagonistic interactions, as implied by a previous study where naturally-exposed individuals were immunized with a candidate PfAMA1 vaccine 39]. It is thus possible that antibodies specific for other antigens may antagonize anti-PfAMA1 responses.

Given the multi-stage nature of *P. falciparum* erythrocyte invasion 40,41], there seem to be two ways in which synergistic antibody action might occur: antibodies of different specificities may act ‘in series’, cumulatively resulting in neutralization via partial inhibition of successive stages of invasion; alternatively, antibodies may act ‘in parallel’, inhibiting a number of closely-related and possibly mutually redundant invasion processes. The precise functions of PfRH5 and other PfRH family members are yet to be fully elucidated. However, it is known that these proteins all bind specific receptors on the erythrocyte and it has been proposed that the PfEBA and PfRH protein families may be involved in tight attachment of the merozoite 41]. PfRH5 cannot be genetically knocked out, and antibodies against basigin can completely eliminate erythrocyte invasion in the absence of antibodies targeting any other ligand-receptor pathways 16]. Therefore, it is unlikely that PfRH5 is functionally redundant with other PfRH proteins, and may instead partner with them to facilitate merozoite invasion of erythrocytes. Further work is necessary to understand how these merozoite ligands interact during erythrocyte invasion, and to elucidate the obviously crucial role of PfRH5 in this process. For now, our results indicate that multi-antigen blood-stage vaccines will be more efficacious when they induce antibodies against antigens with closely-related or complementary ‘parallel’ functions.

It has been previously speculated that one of the major constraints on effective antibody-mediated immunity to the blood stages of *P. falciparum* is the extremely brief window (∼1 minute) of merozoite exposure to antibodies during erythrocyte invasion 42,43]. The duration of exposure of rhoptry and rhoptry-neck antigens such as PfRH5 may be even shorter, as these proteins may not be translocated onto the merozoite surface until after the merozoite contacts the erythrocyte 44]. Our data indeed suggest there is kinetic constraint on antibody-mediated neutralization: the extent of synergy between antibodies was most marked when one antibody targeted the erythrocyte receptor and hence was likely able to reach equilibrium binding.

The inclusion of multiple antigens in a vaccine may incur substantial cost and risk of antigenic competition. It is possible that such effects may well outweigh relatively modest synergistic effects 26]. Consequently, rigorous assessment of possible antigen combinations is required prior to clinical trials. In pre-clinical models, factors other than the presence of synergy must also be considered. These include the quantitative extent of the synergy, the extent of strain-transcending efficacy, and the relative immuno-dominance and possible immune interference arising from different antigen combinations *in vivo*. The lack of clear synergistic effects when antibodies were combined in GIA assays with the FVO parasite clone demonstrates the difficulties in selection of components for multi-antigen vaccines; clearly, for synergistic effects to be apparent in the field, antigens will need to be highly conserved both at the sequence level and in their functional importance to the parasite. While PfRH5 fulfils these criteria, we have yet to identify a second component that in combination with PfRH5 produces truly synergistic, strain-transcending effects.

Furthermore, extrapolating from controlled *in vitro* studies of vaccine-induced rabbit antibodies is more difficult than doing so from studies focusing on drug discovery. Whereas known quantities of drugs can be administered, the quantity and specificity of human antibodies produced against each component of a multi-antigen vaccine cannot be controlled *in vivo* and may vary substantially between individuals; thus, components that synergize when mixed artificially *in vitro* may prove less efficacious *in vivo* due to antigenic interference. Indeed, we found that a 2- to 3-fold increase in anti-PfRH5 IgG is expected to have the same effect as the combination of anti-PfRH4 and anti-PfRH5 IgG without antigenic interference. These data confirm that extremely careful consideration should be given to the prospects for doubling antibody concentrations against a single antigen by improved formulation and/or antigen delivery, rather than attempting to induce consistently high antibody titers against two antigens by co-immunization.

In summary, this study provides further evidence that PfRH5 will be a more effective single-component vaccine than previously-tested merozoite antigens, and encourages its prompt clinical development as a vaccine. We have also provided the clearest demonstration to date that rationally designed antigen combinations may achieve truly synergistic effects of polyclonal antibody against a pathogen. These results point the way towards testing of second-generation PfRH-targeting vaccines, and the identification of additional antigenically-conserved blood-stage antigens that act synergistically with PfRH5.

## Materials and Methods

### Ethics statement

All animal work was approved by the University of Oxford Animal Care and Ethical Review Committee (in its review of the Home Office Project Licence PPL 30/2414), and conducted according to national (U.K. animals scientific procedures act 1986) and international guidelines. Parasite isolates were obtained under a study protocol approved by the Cambodian National Ethics Committee for Health Research and the NIAID Institutional Review Board (ClincalTrials.gov identifier: NCT00341003).

### Viral-vectored vaccine constructs, animals and immunization regimes

The design, production and immunogenicities of the viral-vectored vaccines have been previously described in detail 17]. Briefly, the following antigens were codon-optimized for human expression and synthesized by GeneArt GmbH: PfEBA175 F2 domain (amino acids (aa) 447–795); Pf38 (aa 23–327); PfRAP3 (aa 23–399); PfRH2 (also known as PfRH2a9 45], from the PfRH2a sequence, aa 2030–2531, a sequence shared between PfRH2a and PfRH2b); PfRH4 (aa 28–766, also known as RH4.9 46]); full-length PfRH5 (aa26–526), PfAMA1 (a bivalent construct comprising the ectodomain (aa 24–546) of both the 3D7 and FVO alleles, fused in tandem and linked by a glycine-proline linker, and followed by the transmembrane domain and C-terminus of the FVO allele) 47] and PfMSP1 (a construct containing the conserved blocks of sequence 1, 3, 5 and 12 followed by both of the dimorphic forms of the 42 kDa C-terminus, MSP1_42_, fused in tandem) 48]. For PfEBA175, PfRH2 and PfRH4, constructs were based on fragments known to contain the erythrocyte-binding domains 45,46,49]. Antigens were cloned into the replication-deficient adenovirus human serotype 5 (AdHu5) and the attenuated poxvirus modified vaccinia virus Ankara (MVA) genomes downstream of a mammalian secretory signal (from human tissue plasminogen activator), and the viruses prepared as previously described 17]. All constructs were based on the 3D7 sequence of *P. falciparum*, except for PfEBA175 which was based on the Camp strain of *P. falciparum* 49]. Rabbit immunizations were carried out by Agrobio and Biogenes as previously described 17]. Briefly, female New Zealand white rabbits (2–5 per group) were immunized with 7×10^7^–4.5×10^8^ infectious units of AdHu5 on day 0, and boosted with 5×10^7^–1×10^8^ plaque-forming units MVA on day 56. In the case of the PfRH2 and PfEBA175 groups, rabbits received a third immunization on day 114 (to maximize antibody titers 50,51]) with 100 µg of either PfRH2 or PfEBA175 recombinant protein (produced as described [17]) mixed with 20 µL (18 µg) Abisco adjuvant (ISCOM Matrix M). Serum was collected two weeks after the final immunization.

### Parasite culture and GIA assays

Total IgG was purified from rabbit sera using protein G columns (Pierce). The *P. falciparum* 3D7 and FVO lines were maintained in continuous culture using fresh O^+^ erythrocytes at 2% hematocrit and synchronized either by purification on 65% Percoll gradients followed by incubation in 5% sorbitol, or by magnetic separation (MACS LS columns, Miltenyi Biotech). Synchronized trophozoites were adjusted to 0.5% parasitemia and then incubated for 42 hours (3D7) or 48 hours (FVO) with the various IgG combinations. Final parasitemia was determined by biochemical determination of parasite lactate dehydrogenase 6]. Percentage growth inhibition is expressed relative to wells containing IgG from rabbits immunized with negative controls (vaccines with non-*Plasmodium* antigens) 17]. The anti-PfRH5 mouse mAb QA5 was purified from hybridoma culture supernatant using protein G columns (ADD *et al*., manuscript in preparation). The anti-basigin mAb TRA-1-85 (R&D Systems, UK) was buffer-exchanged into culture medium for use in GIA assays.

GIA assays involving short-term-adapted *P. falciparum* isolates were conducted at the PATH Malaria Vaccine Initiative (MVI) GIA Reference Center as previously described 6], using protein G-purified total IgG from the serum of five rabbits immunized with full-length PfRH5 vaccines and five rabbits immunized with bivalent (3D7+FVO) PfAMA1 vaccines 17]. Cryopreserved stocks of parasite isolates obtained directly from Cambodian patients with uncomplicated malaria in 2009 were adapted to *in vitro* culture for up to 12 weeks. Sequencing and genotyping of PfRH5 in the Cambodian parasite isolates was as previously described 33].

Where EC_50_ was measured for individual rabbit IgG samples, the concentration of IgG required to give 50% GIA (EC_50_) was estimated by interpolation on the plot of log_10_[total IgG] versus % GIA with the measured points connected by straight lines (or extrapolation from the adjacent line segment, in the case of samples for which the EC_50_ value lay outside of the tested IgG concentration range). Very similar results were obtained using non-linear least squares regression, fitting variable slope dose-response curves using the equation %GIA = 100/(1+10∧((log_10_EC_50_-log_10_[IgG])*HillSlope)) (Prism v5.03, GraphPad Software) although the quantity of data for each rabbit IgG sample was not sufficient for reliable curve-fitting (data not shown). Where a single estimate of antigen-specific EC_50_ was calculated for a *P. falciparum* sample by combining data obtained from multiple rabbit IgG samples, a dose-response curve was fitted to all available data by non-linear least squares regression, as above.

### Antigen-specific antibody EC_50_ estimation

The principle of calibration free concentration analysis (CFCA) of antigen-specific antibody by surface plasmon resonance (SPR) has been described elsewhere 31]. We performed CFCA using a Biacore T100 machine, a Biotin CAP chip, and T100 version 2 control and evaluation software (all from GE Lifesciences, Amersham, UK). Running buffer comprising HBS, 3 mM EDTA, 0.05% Tween-20 (‘HBS-EP+’), was prepared and adjusted to pH 7.4, followed by the addition of 1 mg/mL salmon DNA (Sigma) and 2 mg/mL carboxymethyl-dextran (Sigma). The biotin-CAP reagent supplied with the CAP chip was diluted six-fold in HBS-EP+. All experiments were conducted at an analysis temperature of 37°C. PfRH5 and PfAMA1 proteins (enzymatically mono-biotinylated at the C-terminus) were produced by transient transfection of HEK293E cells, as previously described 52], and extensively dialyzed against PBS to remove free biotin. The conformational accuracy of the PfRH5 protein was verified by confirming that the protein bound its receptor, basigin, by SPR [16]; the PfAMA1 protein contains epitopes recognized by sera from naturally-immune African individuals that are destroyed by heat denaturation of the protein (F.H. Osier and CC *et al.*, manuscript in preparation), demonstrating that this protein is also folded correctly.

The measured diffusion coefficient of IgG at 20°C in a solution with the viscosity of water, pH 7.4, is 3.9×10^−11^ m^2^/s 31,53]. The viscosity of the DNA- and dextran-containing buffer at 37°C was 0.754×10^−3^ Pa.s (measured using a TA AR-G2 rheometer [Texas Instruments]). The diffusion coefficient of IgG under the test conditions (37°C) was therefore calculated to be 5.5×10^−11^ m^2^/s. A molecular weight of 150 kDa for IgG was used in the binding model.

Mass-transport limited binding conditions were obtained by capturing a minimum of 800 response units (RU) of antigen. Protein G-purified rabbit IgG samples from five PfRH5-vaccinated rabbits and five PfAMA1-vaccinated rabbits were prepared as above. The same samples were assayed for GIA against 3D7 parasites, and with the exception of one PfAMA1 vaccinated rabbit, these samples were from the same sera used for field isolate assay of GIA (above). IgG samples were diluted 20-fold in *P. falciparum* culture medium, and the total protein concentration measured by spectrometry (Nanodrop, Thermo Scientific). These samples were further diluted 100-fold in running buffer, resulting in final total IgG concentrations in the range 12–25 µg/mL in the samples used for CFCA.

Antigen-specific antibody binding was measured by double reference subtraction, firstly of the binding of antigen-specific antibody to a flow cell coated only with the biotin capture reagent, and secondly of the binding of an equivalent concentration of total IgG from a rabbit immunized with viral vectors lacking a malaria antigen. Initial rates of antigen-specific binding at 5 µL/min and 100 µL/min were measured and compared to permit measurement of concentration and the level of mass-transport limitation (necessary for accuracy of the assay). The chip was regenerated with the manufacturer's supplied regeneration and CAP reagents and fresh antigen prior to each application of antibody; variation in the level of antigen capture between cycles was typically <2%. All results reported were within the instrument manufacturer's recommended quality control parameters, namely initial binding rates in the range 0.3RU to 15 RU/s at 5 µL/min flow, and QC ratio >0.13 (reflecting adequate mass transport limitation for concentration estimation) (Figure S2 and Dataset S1). CFCA data were used to calculate antigen-specific antibody concentrations in the protein G purified total IgG samples used in assays of GIA. By combining this with the GIA EC_50_ in terms of total IgG measured as above, the GIA EC_50_ in terms of antigen-specific antibody was calculated.

### Assessment of synergy

Methods that have been widely used for assessing combinations of drugs were applied here to the assessment of synergy between antibodies of differing antigen specificities. Synergy between two agents is defined as an effect greater than would be predicted from the two agents, when mixed, acting independently of each other to produce an additive effect 35]. An effect less than that predicted from two agents acting independently may be termed antagonism, although it may be worth drawing a distinction between a sub-additive combination (in which the effect remains at least as strong as that of the more potent individual component) and an antagonistic combination in which the two agents together actually perform worse than at least one agent would have performed alone. Although conceptually relatively straightforward, there is a degree of controversy regarding these definitions, stemming primarily from disagreement regarding the appropriate method of calculation of the expected additive effect of two agents. As has been reviewed elsewhere, there are multiple differing definitions of pharmacological additivity: this work employs two such definitions, each of which have properties which render them useful in some contexts 35].

Bliss' definition of independent action (henceforth referred to as ‘Bliss additivity’) is related to probability theory 34]. In the context of neutralization of a population of merozoites which would otherwise have invaded erythrocytes, suppose two antisera (A+B) each individually have a probability of neutralizing a given merozoite, and then define the probability of *successful* invasion in the presence of A as P(Inv_A_), or in the presence of B as P(Inv_B_). If A+B are Bliss additive, the probability of *successful* invasion in the presence of a mixture of *both* A *and* B, P(Inv_A+B_) will be the same as that of the occurrence of *both* of two independent events: P(Inv_A+B_) = P(Inv_A_∩Inv_B_) = P(Inv_A_).P(Inv_B_).

Malaria vaccinologists conventionally express neutralization in terms of percentage GIA = (1-probability of *successful* invasion)*100, hence P(Inv_A_) = 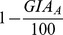. Rearranging gives rise to the following equation for Bliss additivity in GIA:
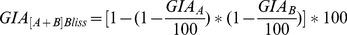
(1)


Bliss' definition of additivity has the advantage that a prediction of an additive effect only requires knowledge of the level of effect of each individual constituent of a mixture. We therefore used this definition to screen combinations of anti-PfRH5 (‘antibody A’) and antibodies against each of seven other antigens (‘antibody B’) for synergistic activity. In this screening assay, we measured the GIA effect of a range of concentrations of antibodies against antigen B with and without the addition of a fixed concentration of anti-PfRH5. We compared the observed effect GIA_[RH5+B] Obs_ to the expected effect GIA_[RH5+B] Bliss_, calculated using Equation 1 and the observed effect of the single concentration of anti-PfRH5 and the observed effect GIA_B_ at each tested concentration. Statistically significant deviations between the observed and predicted values indicated non-independence, either synergy (greater GIA effect than the predicted value) or sub-additivity (weaker effect than the predicted value).

Bliss' definition of additivity, though appealingly simple to apply, does not account for the fact that the shapes of different agents' concentration *vs.* effect curves differ: an agent with a rapidly steepening concentration *vs.* effect curve is said to be positively co-operative. Mixtures of such an agent *with itself* may thus appear to be synergistic according to Bliss' definition (in different circumstances, a misleading apparent antagonistic effect could also arise). A more complex but more robust definition of additive action, avoiding this problem, is that of Loewe additivity 35]. When an experiment is conducted with an appropriate selection of mixtures of concentrations of agents A and B, it is possible to plot a three-dimensional plot (or contour plot) of [A] versus [B] versus effect. We performed such GIA experiments for two combinations of antibody (PfRH5 with either PfRH4 or PfAMA1), using all possible combinations of six concentrations of both antibodies ranging from 0 to 5 mg/mL total IgG. From these 3-dimensional data, a two dimensional plot on axes of [A] and [B] can be constructed in which a line links points (A,B) at which the mixture [A]+[B] results in the same level of effect (an ‘isobologram’). We constructed such plots illustrating mixtures of antibody which achieved 50% GIA. Total IgG EC_50_ values were 0.625 or 1.25 mg/mL for anti-PfRH5 IgG alone, and 2.5 mg/mL for anti-PfAMA1 IgG alone – these were within the range of previously measured values (Table 1). For anti-PfRH4 alone, the concentration of IgG used in these experiments was insufficient to give 50% GIA. Therefore, a concentration of 10 mg/mL, which had previously been shown to give 50% GIA using the same purified IgG 17], was assumed as the EC_50_ value for anti-PfRH4.

The axes of such a plot can be labelled either with simple concentrations, or with concentration of each agent as proportion of its EC_50_ (i.e. 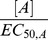).

Loewe's definition of additivity states that, for such a plot:
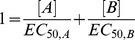
(2)


In other words, if agents A+B are Loewe additive, a 50% effect will be achieved by a mixture of any percentage of the EC_50_ of A and any percentage of the EC_50_ of B, such that the two percentages sum to 100%. On the isobologram plot, such a combination would result in a straight line between x = EC_50,A_ and y = EC_50,B_. Mixtures which achieve synergistic effects will require lower concentrations to achieve 50% effect, resulting in a concave isobologram.

This approach has the additional benefit of permitting quantification of the strength of synergistic (or antagonistic) action between two agents. Hewlett's synergy index, S 35] measures the degree of concavity of the isobologram, with the magnitude of S above 1 implying the level of synergy: 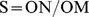
(3)where, ON = distance from origin to intersection of the Loewe additivity line and the x = y line, and OM = distance from origin to intersection of 50% isobologram and the x = y line.

### Statistical analysis

Differences in EC_50_ values between anti-PfRH5 and anti-PfAMA1 IgG were determined by Mann-Whitney U test. Where EC_50_ values were estimated by regression, differences in EC_50_ and the slope of the curve were simultaneously assessed by extra sum-of-squares F-test. Differences between the observed GIA for each combination of antibodies and the predicted GIA based on Bliss independence were determined by repeated measures two-way ANOVA with Bonferroni post-hoc testing. *P* values of <0.05 were considered statistically significant. All analyses were conducted using GraphPad Prism version 5.03 for Windows (GraphPad Software Inc., USA).

## Supporting Information

Dataset S1 **Output from CFCA model-fitting, including initial binding rates at low and high flow rates and QC ratios.**
(XLSX)

Figure S1 **GIA assays with total purified IgG against PfRH5 and PfAMA1 using short-term-adapted parasites from Cambodia.** At each concentration tested, anti-PfRH5 total IgG achieved a greater degree of growth inhibition than total IgG from rabbits vaccinated with bivalent (3D7+FVO) PfAMA1. **Panels A–E** illustrate GIA results obtained against Cambodian parasite isolates CP803, CP806, CP830, CP845 and CP887 respectively, with IgG from each of 10 rabbits. All samples were tested at 0.31, 1.25 and 5 mg/mL. Each point illustrates the mean of triplicate wells; lines link results for a single rabbit. Red lines indicate results for PfRH5-vaccinated rabbits; black lines indicate results for PfAMA1-vaccinated rabbits. **Panel F** summarizes the data presented in panels **A–E** in terms of EC_50_ values for each sample against each parasite isolate, with each red point indicating a PfRH5-vaccinated rabbit and each black point indicating a PfAMA1-vaccinated rabbit. Horizontal dotted lines indicate the upper and lower IgG concentrations tested; median EC_50_ values outside this range were calculated by extrapolation from these data and hence should be regarded as approximations only.
(TIF)

Figure S2 **Example of CFCA data processing.** In each panel, green line represents responses with test sample at flow-rate of 100 µL/min, red line represents responses with test sample at 5 µL/min, whilst the grey lines represent equivalent responses with blank samples (IgG from non-immunized rabbits). x-axis represents time (spanning total of 80 seconds, of which period of sample injection = 35 seconds). **Panel A**: Responses on PfRH5-coated flow cell. **Panel B**: Responses after subtraction of non-PfRH5-coated reference cell. **Panel C**: Test sample responses after subtraction of blank-sample responses. It can be seen that this early phase of the antibody-binding reaction proceeds at a linear rate. The accelerated rate of the reaction under high-flow conditions indicates that the reaction is partially mass-transport limited under slow-flow conditions, permitting accurate extrapolation to rate of reaction under absolute mass-transport limitation, and hence to concentration.
(TIF)
