# Supplementary figures and images for: Enhancing Blockade of Plasmodium falciparum Erythrocyte Invasion: Assessing Combinations of Antibodies against PfRH5 and Other Merozoite Antigens
Source: PLoS Pathog. 2012 Nov 8;8(11):e1002991. doi: 10.1371/journal.ppat.1002991 (PMC3493472; doi:10.1371/journal.ppat.1002991)

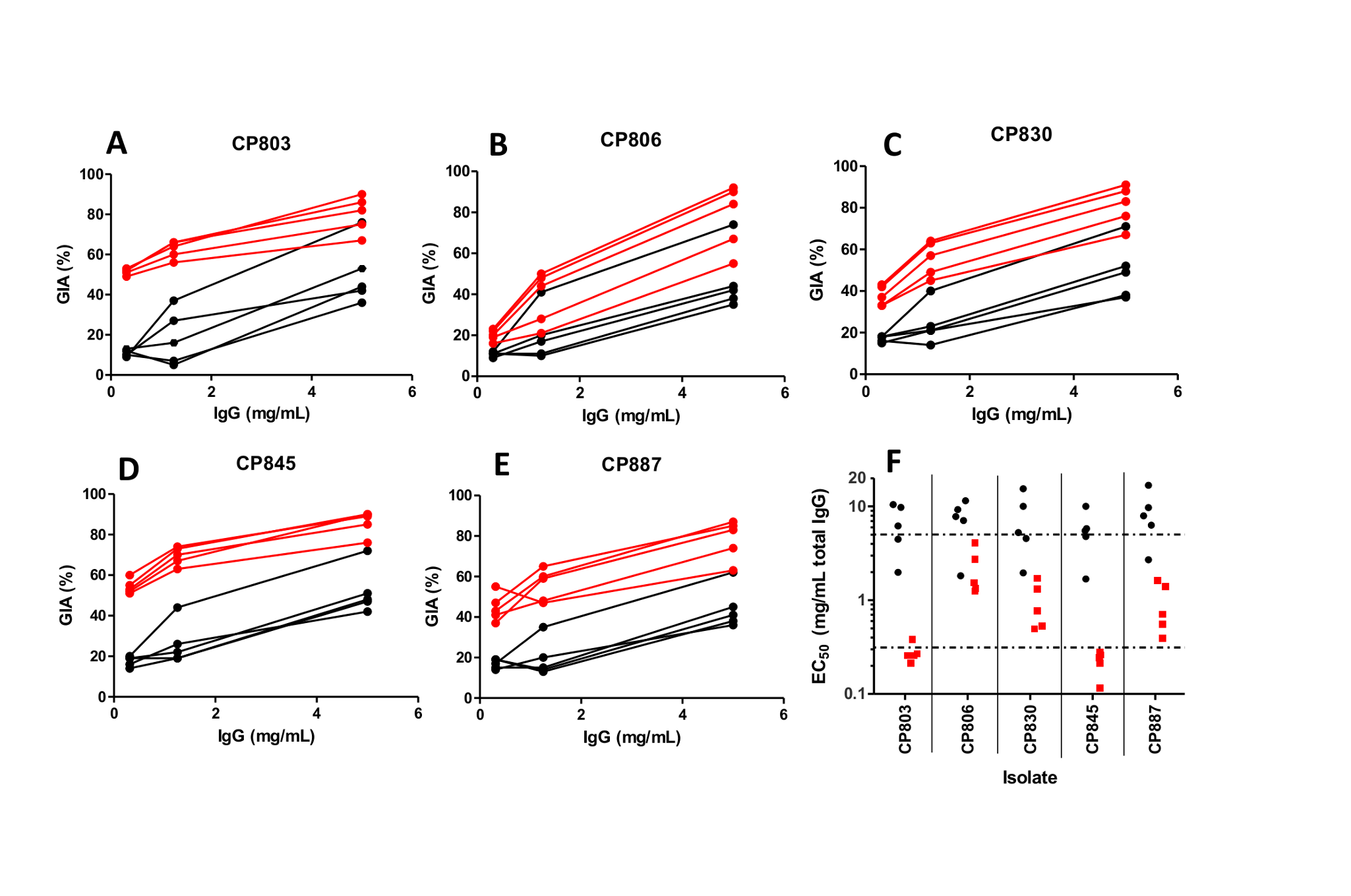

Supplement: Figure S1 — GIA assays with total purified IgG against PfRH5 and PfAMA1 using short-term-adapted parasites from Cambodia. At each concentration tested, anti-PfRH5 total IgG achieved a greater degree of growth inhibition than total IgG from rabbits vaccinated with bivalent (3D7+FVO) PfAMA1. Panels A–E illustrate GIA results obtained against Cambodian parasite isolates CP803, CP806, CP830, CP845 and CP887 respectively, with IgG from each of 10 rabbits. All samples were tested at 0.31, 1.25 and 5 mg/mL. Each point illustrates the mean of triplicate wells; lines link results for a single rabbit. Red lines indicate results for PfRH5-vaccinated rabbits; black lines indicate results for PfAMA1-vaccinated rabbits. Panel F summarizes the data presented in panels A–E in terms of EC50 values for each sample against each parasite isolate, with each red point indicating a PfRH5-vaccinated rabbit and each black point indicating a PfAMA1-vaccinated rabbit. Horizontal dotted lines indicate the upper and lower IgG concentrations tested; median EC50 values outside this range were calculated by extrapolation from these data and hence should be regarded as approximations only. (TIF) [file ppat.1002991.s002.tif]

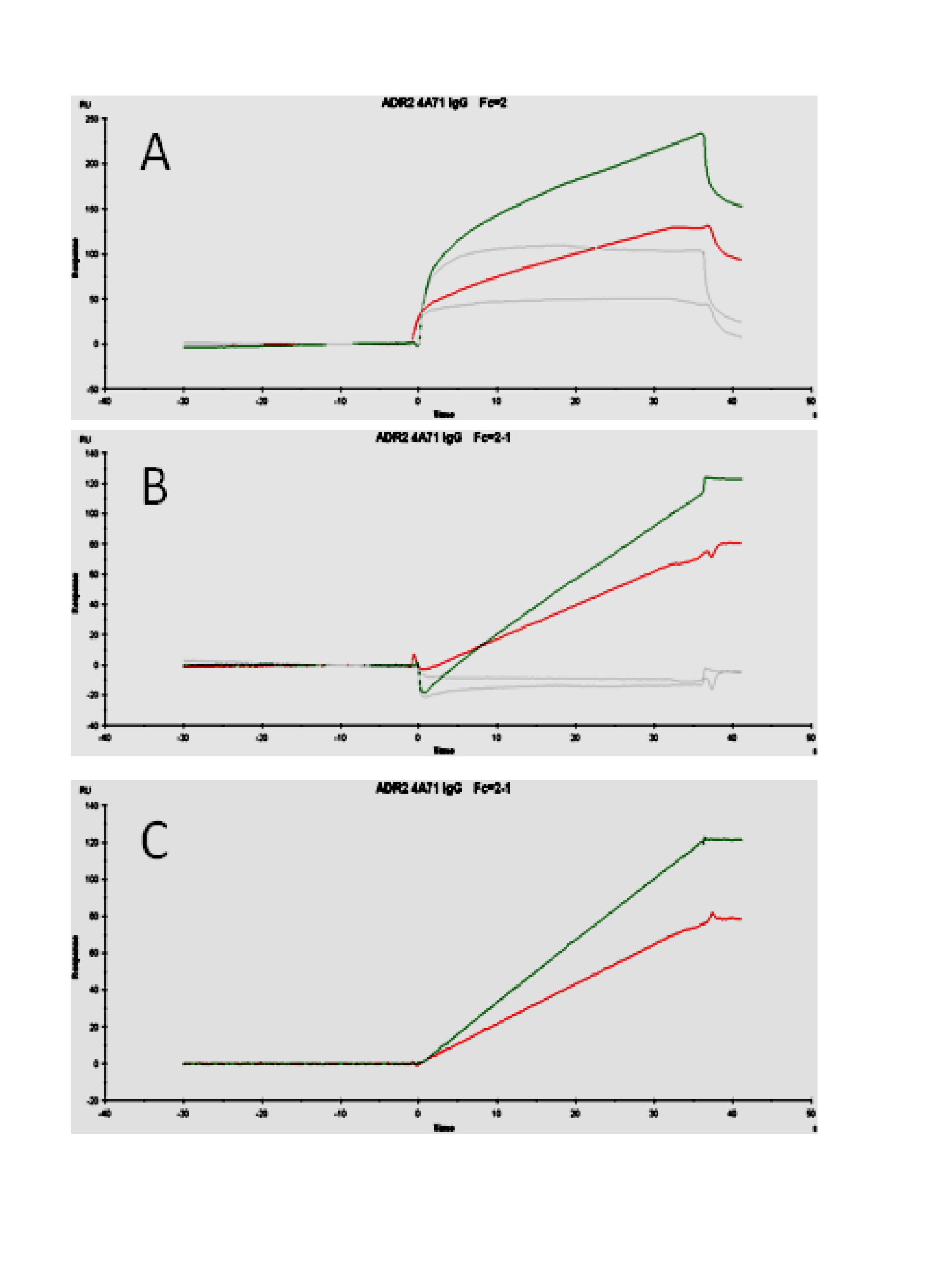

Supplement: Figure S2 — Example of CFCA data processing. In each panel, green line represents responses with test sample at flow-rate of 100 µL/min, red line represents responses with test sample at 5 µL/min, whilst the grey lines represent equivalent responses with blank samples (IgG from non-immunized rabbits). x-axis represents time (spanning total of 80 seconds, of which period of sample injection = 35 seconds). Panel A: Responses on PfRH5-coated flow cell. Panel B: Responses after subtraction of non-PfRH5-coated reference cell. Panel C: Test sample responses after subtraction of blank-sample responses. It can be seen that this early phase of the antibody-binding reaction proceeds at a linear rate. The accelerated rate of the reaction under high-flow conditions indicates that the reaction is partially mass-transport limited under slow-flow conditions, permitting accurate extrapolation to rate of reaction under absolute mass-transport limitation, and hence to concentration. (TIF) [file ppat.1002991.s003.tif]
